# The occurrence of some foodborne pathogens recovered from poultry meat in Shahrekord, Iran

**DOI:** 10.5455/javar.2023.j670

**Published:** 2023-06-30

**Authors:** Sayed Ahmad Nourbakhsh, Ebrahim Rahimi

**Affiliations:** Department of Food Hygiene and Public Health, Shahrekord Branch, Islamic Azad University, Shahrekord, Iran.

**Keywords:** *Arcobacter butzleri*, *Listeria monocytogenes*, *Campylobacter jejuni*, *Staphylococcus aureus*, poultry meat, occurrence

## Abstract

**Objective:**

*Arcobacter butzleri*, *Listeria monocytogenes*, *Staphylococcus aureus*, and *Campylobacter* *jejuni* are significant foodborne pathogens regarding the consumption of raw poultry meat. An existing survey was conducted to assess the occurrence of *S. aureus*, *C. jejuni*, *A. butzleri*, and *L. monocytogenes* in raw poultry meat samples.

**Materials and Methods:**

Ninety-four raw ostrich, turkey, chicken, and quail meat samples were collected and subjected to culture-based analysis. *Staphylococcus aureus*, *C. jejuni*, *A. butzleri*, and *L. monocytogenes* isolates were confirmed by standard biochemical techniques.

**Results:**

The occurrence of *A. butzleri*, *C. jejuni*, *L. monocytogenes*, and *S. aureus* in poultry meat samples was 11.45%, 17.70%, 1.04%, and 16.66%, respectively.* L. monocytogenes* was absent in chicken, turkey, and ostrich meat samples. Only one quail meat (4.16%) was positive for *L. monocytogenes*. The uppermost contamination rate with *A. butzleri*, *C. jejuni*, and *S. aureus* was found in chicken (25%), turkey (25%), and turkey (25%) meat samples, respectively. The concurrent occurrence of *A. butzleri* + *C. jejuni* + *S. aureus* bacteria amid the examined poultry meat samples was 2.08%.

**Conclusion:**

This is an initial report of *A. butzleri*, *S. aureus,* *C. jejuni*, and *L. monocytogenes* in poultry meat samples. Adequate cooking of poultry meat can diminish foodborne diseases due to *A. butzleri*, *S. aureus*, *L. monocytogenes*, and *C. jejuni *bacteria, and these species may constitute a public health problem.

## Introduction

Poultry meat is an excellent source of many minerals and vitamins. The human contribution to poultry meat inspection and purchase increased the probability of bacterial contamination and complicated foodborne diseases [1].

Some pathogens source the greatest cases of foodborne infection, e.g., *Campylobacter*, *Salmonella*, *Escherichia*, *Arcobacter*, *Listeria*, and others [2–5]. *Staphylococcus aureus* (*S. aureus*) is a Gram-positive bacterium that originates from the respiratory tract and on the skin and handles unadorned nosocomial and community-acquired infections and foodborne diseases [3]. Many gastrointestinal disorders are accredited to *S. aureus *.

*Listeria monocytogenes *(*L. monocytogenes*) is a Gram-positive bacterium that can cause severe diseases, including meningoencephalitis, septicemia, mastitis, metritis, keratoconjunctivitis, iritis, and abortion in humans and animals, principally distressing newborn, pregnant, and immunocompromised persons [4]. It is also responsible for the occurrence of foodborne diseases recognized by fever and, sometimes, fetal abortion in both humans and animals [4].

*Campylobacter* species are Gram-negative bacteria and are considered the most common gastroenteritis. *Campylobacter jejuni *(*C. jejuni*) is this genus’s most significant bacterium, accompanied by human diseases [5]. Clinical manifestations of campylobacteriosis comprise diarrhea, abdominal cramp, and fever. Bloody diarrhea may be present in complicated cases. The bacterium is also responsible for Guillain–Barré Syndrome, recognized with immune-mediated neuropathies, ataxia, areflexia, and ophthalmoplegia [5].

*Arcobacter* species are Gram-negative bacteria of the *Campylobacter*aceae family. *Arcobacter butzleri *(*A. butzleri*) is one of the most important species in the genus *Arcobacter*. *Arcobacter butzleri* has developed as a significant foodborne zoonotic bacterium responsible for diverse clinical syndromes [6].

Poultry meat and its products are measured as reservoirs of *S. aureus*, *C. jejuni*, *A. butzleri*, and *L. monocytogenes* [7,8]. Poultries and infected staff may transport *S. aureus*, *C. jejuni*, *A. butzleri,* and *L. monocytogenes* [7,8]. Thus, assessing these pathogenic foodborne bacteria in poultry meat is indispensable.

Alterations in recovery rates of food contamination between some studies on poultry products from different countries have been reported [1,2,7–9]. To our knowledge, work has yet to address the prevalence of *S. aureus*, *C. jejuni*, *A. butzleri*, and *L. monocytogenes* in poultry meat in Iran. There is inadequate data to rule out the occurrence of *S. aureus*, *C. jejuni*, *A. butzleri*, and *L. monocytogenes* in poultry meat in Iran. Thus, a current survey was done to measure the occurrence of *S. aureus*, *C. jejuni*, *A. butzleri*, and *L. monocytogenes* in ostrich, turkey, chicken, and quail meat samples collected from Shahrekord, Iran.

## Materials and Methods

### Ethical approval

The survey rendered the procedure accepted by Islamic Azad University, Shahrekord Branch, Shahrekord, Iran.

### Samples

From May to August 2019, 94 raw poultry meat samples, including ostrich (*n =* 24), turkey (*n =* 24), chicken (*n =* 24), and quail (*n =* 24), were collected from the retail centers of Shahrekord, Iran. Sampling was done from the femur muscles of poultry. The 40 gm of samples in sterile glass bottles) were rapidly transferred to the laboratory at 4°C.

### Isolation and identification of S. aureus

Isolation was performed on the 25 gm of samples. Isolation criteria were done according to the method labeled beforehand. Samples were primarily homogenized with 225 ml of buffered peptone water (Merck, Germany) using Stomacher (Interscience, Saint-Nom, France). Afterward, samples (5 ml) were added to Trypticase Soy Broth (50 ml) (Merck, Germany) containing NaCl (10%) and sodium pyruvate (1%). Incubation was done at 35°C for 18 h. Afterward, a loopful of culture was transferred into Baird-Parker agar (Merck, Germany) containing egg yolk tellurite emulsion. Incubation was carried out for 24 h at 37°C. *S. aureus* identification was performed by biochemical tests [10].

### Isolation and identification of L. monocytogenes

A similar volume of samples was primarily homogenized with 225 ml of *Listeria* enrichment broth (Merck, Germany). Incubation was carried out for 24 h at 37°C. Afterward, a milliliter of samples was transferred to Frazer broth (9 ml) (Merck, Germany). Incubation was carried out for 24 h at 37°C. PALCAM agar (Merck, Germany) and Oxford agar (Merck, Germany) media were used for other enrichment. Incubation was done for 48 h at 35°C. Three black colonies with black sunken were cultured on Tryptone Soy agar (Merck, Germany) containing yeast extract (0.6%). Incubation was carried out for 24 h at 37°C. *Listeria monocytogenes* was identified by biochemical tests [11].

### Isolation and identification of C. jejuni

A similar volume of samples was primarily homogenized with 225 ml of Preston enrichment broth (HiMedia, India) containing sheep blood (5%) and *Campylobacter* selective supplement IV (HiMedia, India). Microaerophilic incubation was done for 24 h at 42°C. Afterward, the enrichment (0.1 ml) was added to *Campylobacter* selective agar (HiMedia, India), which contained a specific antibiotic (HiMedia, India) and sheep blood (5%) [12].

### Isolation and identification of A. butzleri

A similar volume of samples was primarily homogenized with 225 ml of *Arcobacter* broth containing trimethoprim (64 mg/l), amphotericin B, novobiocin (32 mg/l), cefoperazone, 5-fluorouracil (100 mg/l), and teicoplanin (Oxoid, UK). Incubation was done for 48 h at 30°C. Afterward, bacteria were transferred to *Arcobacter* agar (Oxoid, UK) containing the same ingredients as above and sheep blood. Incubation was done for 48 h at 30°C. *Acobacter butzleri* was identified by biochemical tests [13].

### Numerical examination

A numerical examination was performed using SPSS 21.0 (Chicago, USA). The chi-square and Fisher’s exact two-tailed tests were performed to analyze the data. *p* < 0.05 was determined as a numerically significant level.

## Results

The current research assessed the occurrence of *S. aureus*, *C. jejuni*, *A. butzleri*, and *L. monocytogenes* in ostrich, turkey, chicken, and quail meat samples. Table 1 indicates the occurrence of foodborne bacteria in the studied poultry meat samples. The occurrence of *A. butzleri*, *C. jejuni*, *L. monocytogenes*, and *S. aureus* in examined poultry meat samples was 11.45%, 17.70%, 1.04%, and 16.66%, respectively. The technique failed to detect any *L. monocytogenes* bacteria in the ostrich, turkey, and chicken meat samples. Additionally, only one quail sample (4.16%) was positive for *L. monocytogenes*. The highest *A. butzleri* was found in chicken meat samples (25%). The highest occurrence of *C. jejuni* was found in turkey meat samples (25%). The highest occurrence of *S. aureus* was found in turkey meat samples (25%). A statistically significant difference was obtained between the type of poultry meat samples and bacteria (*p* < 0.05).

**Table 1.** Occurrence of foodborne bacteria in numerous kinds of poultry meat samples.

Types of meat samples	N samples collected	No. of samples positive for bacteria (%)			
		*Arcobacte. butzleri*	*Campylobacter jejuni*	*Listeria monocytogenes*	*Staphylococcus aureus*
Chicken	24	6 (25)	8 (33.33)	-	4 (16.66)
Turkey	24	3 (12.50)	6 (25)	-	6 (25)
Ostrich	24	1 (4.16)	1 (4.16)	-	3 (12.50)
Quail	24	1 (4.16)	2 (8.33)	1 (4.16)	3 (12.50)
Total	96	11 (11.45)	17 (17.70)	1 (1.04)	16 (16.66)

**Table 2.** Combined occurrence of foodborne bacteria amid the poultry meat samples.

Types of meat samples (N. samples)	No of samples positive for bacteria (%)						
	*Arcobacter butzleri + Campylobacter jejuni*	*Arcobacter butzleri + Listeria Monocytogenes*	*Arcobacter butzleri + Staphylococcus aureus*	*Campylobacter jejuni + Listeria monocytogenes*	*Campylobacter jejuni + Staphylococcus aureus*	*Listeria monocytogenes + Staphylococcus aureus*	*Arcobacter butzleri + Campylobacter jejuni + Staphylococcus aureus*
Chicken (24)	3 (12.50)	-	2 (8.33)	-	1 (4.16)	-	1 (4.16)
Turkey (24)	1 (4.16)	-	1 (4.16)	-	2 (8.33)	-	1 (4.16)
Ostrich (24)	-	-	1 (4.16)	-	1 (4.16)	-	-
Quail (24)	-	-	-	1 (4.16)	-	1 (4.16)	-
Total (96)	4 (4.16)	-	4 (4.16)	1 (1.04)	4 (4.16)	1 (1.04)	2 (2.08)

Table 2 indicates the combined occurrence of foodborne bacteria among the poultry meat samples. Total occurrence of *A. butzleri* + *C. jejuni*, *A. butzleri* + *S. aureus*, *C. jejuni* + *L. monocytogenes*, *C. jejuni* + *S. aureus*, *L. monocytogenes* + *S. aureus*, and *A. butzleri* + *C. jejuni* + *S. aureus* bacteria amid the examined poultry meat samples was 4.16%, 4.16%, 1.04%, 4.16%, 1.04%, and 2.08%, respectively. The applied method failed to detect *A. butzleri* + *L. monocytogenes* bacteria in all poultry meat samples. Chicken meat samples had the uppermost occurrence of *A. butzleri* + *C. jejuni* (12.50%). Chicken meat samples had the highest *A. butzleri* + *S. aureus* (8.33%). Turkey meat samples had the highest *C. jejuni* + *S. aureus* (8.33%). A statistically significant difference was obtained between poultry meat samples and the combined occurrence of bacteria (*p* < 0.05).

## Discussion

Foodborne diseases in the United States cause yearly, about 75 million diseases, 300,000 hospitalizations, and 6,000 deaths [14]. *Staphylococcus aureus*, *C. jejuni*, *A. butzleri*, and *L. monocytogenes* are essential pathogens in gastrointestinal, foodborne, and nosocomial infections [15]. *Staphylococcus aureus* is a significant cause of foodborne diseases, causing an estimated 240,000 diseases in the United States [14,15]. The yearly number of described cases of foodborne diseases through *Campylobacter* species in the European Union was 225,000 in 2011. However, it could be as high as 10 million a year [16]. In the United States, *L. monocytogenes* is an imperative foodborne pathogen contributing to 1,700 foodborne diseases, 1,500 hospitalizations, and 250 deaths [17]. *Arcobacter* species, especially *A. butzleri*, is associated with several outbreaks of foodborne diseases globally [18].

The current investigation assessed the occurrence of *S. aureus*, *C. jejuni*, *A. butzleri*, and *L. monocytogenes* bacteria amid the raw ostrich, turkey, chicken, and quail meat samples collected from Shahrekord, Iran. Outcomes revealed that the bacteria of* S. aureus*, *C. jejuni*, *A. butzleri*, and *L. monocytogenes* in the examined raw poultry meat samples were 16.66%, 17.70%, 11.45%, and 1.04%, respectively. Thus, *C. jejuni* had the highest occurrence. Additionally, the uppermost occurrence of *A. butzleri*, *C. jejuni*, *L. monocytogenes*, and *S. aureus* was found in chicken meat (25%), chicken meat (33.33%), quail meat (4.16%), and turkey meat (25%). The high occurrence of bacteria in examined poultry meat samples may be due to the primary presence of bacteria in studied poultry, the occurrence of cross-contamination through diverse stages of the slaughter, the transmission of bacteria from infected staff and workers to poultry carcasses, and finally, the transmission of bacteria from contaminated water used in the washing of poultry carcasses. Diverse research has been conducted in this field.

 The subordinate occurrence of *S. aureus* was conveyed in foodstuff from Italy [19] and the Netherlands [20]. The occurrence of *S. aureus* in meat in Brazil [21], Egypt [22], Germany [23], and Denmark [24] were 21.72%, 40.80%, 71.50%, and 52.00%, respectively. Moreover, the role of poultry meat as a reservoir of *S. aureus* bacteria was also recognized in samples collected from Australia [25], the United Kingdom [26], and the United States [27]. Our findings unveiled that turkey and chicken meat samples may be significant reservoirs of *S. aureus* bacteria.

Poultry is an imperative reservoir of *A. butzleri* [28–30]. It is considered a source of infection transmission and spread [28,29]. Poultry intestines have been projected to harbor *Arcobacter* and contaminate the slaughterhouses throughout carcass processing, thereby preventing additional contamination. The occurrence of *Arcobacter* in chicken meat samples collected from Iran [31], Nigeria [32], Malaysia [33], The Netherlands [34], Korea [35], and Spain [28] was 37.50%, 32.00%, 39.20%, 0%–100%, 23.20%, and 64.30%, respectively. Rahimi [29] conveyed that the occurrence of *Arcobacter* strains amid raw chicken, turkey, quail, partridge, duck, ostrich, and geese meat samples were 28.00%, 11.00%, 12.00%, 7.50%, 11.40%, 3.30%, and 8.00%, respectively. He revealed that 90.10% of *Arcobacter* isolates were related to *A. butzleri*. de Oliveira et al. [36] conveyed that the occurrence of *Arcobacter* spp. in chicken meat samples was 18.30%, in which 63.60% of isolates were related to *A. butzleri*. Molva and Atabay [37] conveyed that the occurrence of *Arcobacter* species amid the chicken meat samples was 55%, in which 80% of isolates were *A. butzleri*.

*Listeria monocytogenes* has a lower occurrence in comparison with other tested bacteria. Perković et al. [38] specified that the occurrence of *L. monocytogenes* among the poultry meat samples was 1%. Turkey meat was the most commonly contaminated (2%). Osaili et al. [39] specified that *L. monocytogenes* was recovered from 9.40% of raw broiler samples, higher than our findings. Meyer et al. [40] signified that the *Listeria* species were isolated from 6% of poultry meat product samples, which was also higher than our findings. In keeping with this, various occurrence rates of *L. monocytogenes* have been reported worldwide, ranging from 8% to 99% [39,41,42]. Schäfer et al. [43] described the contamination rate of raw chicken meat samples as 44.19%. Bilir Ormanci et al. [44] conveyed that the occurrence of *L. monocytogenes* in turkey meat samples was 12.70% [44].

*Campylobacter jejuni* is well adapted to growth and survival in poultry meat and dependent products. Mezher et al. [45] found that the occurrence of *Campylobacter *species in chicken meat samples were 6.80%, and 27.27% of isolates were identified as *C. jejuni*. The whole occurrence of *C. jejuni* amid the poultry meat samples collected from Iraq [46], Iran [47], Pakistan [48], India [49], Korea [50], and China [51] was 10%, 6.84%, 40%, 26.30%, 36.30%, and finally 1.82% to 56.00%, respectively. Boost occurrence of *Campylobacter* species was also conveyed in poultry meat samples collected from European countries (29%–41%) [52–55].

As observed, diverse research reveals various occurrence rates of bacteria. The variance in data suggests that season, time and place of sampling, method of sampling, types of samples, and even laboratory methods applied in research may affect the outcomes of an occurrence rate. Different hygienic levels of poultry flocks and shopping centers may affect bacteria in diverse investigations.

## Conclusions

An existing survey assessed the occurrence of *S. aureus*, *C. jejuni*, *A. butzleri*, and *L. monocytogenes* foodborne bacteria in raw ostrich, turkey, chicken, and quail meat samples. The occurrence of *A. butzleri*, *C. jejuni*, *L. monocytogenes*, and *S. aureus* in examined poultry meat samples was 11.45%, 17.70%, 1.04%, and 16.66%, respectively, which was notable. Furthermore, chicken and turkey had a higher contamination rate. Some samples were simultaneously contaminated with two or three foodborne bacteria, demonstrating the boosted importance of poultry meat as a reservoir of pathogenic bacteria. An existing survey is an initial report of *A. butzleri*, *C. jejuni*, *L. monocytogenes*, and *S. aureus* in ostrich, turkey, chicken, and quail meat samples. Our findings revealed that only quail meat was a reservoir for *L. monocytogenes*. Appropriate timing and adequate temperature for poultry meat samples can reduce the risk of transmission of *A. butzleri*, *C. jejuni*, *L. monocytogenes*, and *S. aureus* bacteria to humans. Nevertheless, supplementary surveys can determine additional information regarding *A. butzleri*, *C. jejuni*, *L. monocytogenes*, and *S. aureus* bacteria in poultry meat.

## List of Abbreviations

*A. butzleri*,* Arcobacter butzleri; C. jejuni*,* Campylobacter jejuni*; *L. monocytogenes*,* Listeria monocytogenes; S. aureus*,* Staphylococcus aureus*
